# Deletion of *PDK1* Causes Cardiac Sodium Current Reduction in Mice

**DOI:** 10.1371/journal.pone.0122436

**Published:** 2015-03-17

**Authors:** Zhonglin Han, Yu Jiang, Yuqing Yang, Xuehan Li, Zhongzhou Yang, Kejiang Cao, Dao W. Wang

**Affiliations:** 1 Department of Cardiology, the First Affiliated Hospital of Nanjing Medical University, Nanjing, China; 2 Department of Geriatrics, the First Affiliated Hospital of Nanjing Medical University, Nanjing, China; 3 Ministry of Education Key Laboratory of Model Animal for Disease Study, Model Animal Research Center, Nanjing University, Nanjing, China; Xuzhou Medical College, CHINA

## Abstract

**Background:**

The AGC protein kinase family regulates multiple cellular functions. 3-phosphoinositide-dependent protein kinase-1 (*PDK1*) is involved in the pathogenesis of arrhythmia, and its downstream factor, Forkhead box O1 (Foxo1), negatively regulates the expression of the cardiac sodium channel, Nav1.5. Mice are known to die suddenly after *PDK1* deletion within 11 weeks, but the underlying electrophysiological bases are unclear. Thus, the aim of this study was to investigate the potential mechanisms between *PDK1* signaling pathway and cardiac sodium current.

**Methods and Results:**

Using patch clamp and western blotting techniques, we investigated the role of the *PDK1*-Foxo1 pathway in *PDK1* knockout mice and cultured cardiomyocytes. We found that *PDK1* knockout mice undergo slower heart rate, prolonged QRS and QTc intervals and abnormal conduction within the first few weeks of birth. Furthermore, the peak sodium current is decreased by 33% in cells lacking *PDK1*. The phosphorylation of Akt (308T) and Foxo1 (24T) and the expression of Nav1.5 in the myocardium of *PDK1*-knockout mice are decreased, while the nuclear localization of Foxo1 is increased. The role of the *PDK1*-Foxo1 pathway in regulating Nav1.5 levels and sodium current density was verified using selective *PDK1*, Akt and Foxo1 inhibitors and isolated neonatal rat cardiomyocytes.

**Conclusion:**

These results indicate that *PDK1* participates in the dysregulation of electrophysiological basis by regulating the *PDK1*-Foxo1 pathway, which in turn regulates the expression of Nav1.5 and cardiac sodium channel function.

## Introduction

3-phosphoinositide-dependent protein kinase-1 (*PDK1*), a key member of the AGC (protein kinase A, protein kinase G and protein kinase C) protein kinase family, acts as an upstream protein kinase by phosphorylating and activating many other AGC-family members, including protein kinase B (PKB)/Akt [1], p70 ribosomal S6 kinase (S6K) [2], serum and glucocorticoid-induced protein kinase (SGK) [3], and Forkhead box O (Foxo) [4]. *PDK1* has an established role in regulating physiological processes relevant to metabolism, growth, proliferation and survival [5]. Moreover, *PDK1* [6] and its upstream or downstream factors, including phosphatidyl inositol 3-kinase (PI3K) [7], Akt and mTOR [8], are involved in heart failure and pathologic heart remodeling. Mice with *PDK1* deletion have markedly reduced myocardium, smaller cardiomyocytes, thinner ventricles and enlarged atria, which eventually leads to heart failure and sudden death within 11 weeks [9]; however, the underlying mechanisms of abnormal electrophysiological basis related deaths are not clear.

Recently, the involvement of the AGC protein kinase family in regulating arrhythmia has drawn considerable attention. PI3K signaling is associated with the alteration of ion channel function, which is established to play a role in the development of cardiac arrhythmia. Drug-induced increases in action potential and QT prolongation induce inhibition in multiple ion currents, including peak Na+ current, and are accompanied by decreased PI3K signaling [10]. Foxo1 transcription factor, one of the major PI3K Akt downstream effectors, binds to promoter sequences to regulate the expression of target genes, including the sodium channel gene *SCN5A*. Akt negatively regulates Foxo1 by phosphorylation on Thr24, Ser256 and Ser319, which induces Foxo1 to bind to 14-3-3, and consequently triggers the relocalization of Foxo1 from the nucleus to the cytoplasm, where it becomes inactivated [4]. Foxo1 inhibits the expression of Nav1.5, the protein encoded by *SCN5A*, upon treatment with hydrogen peroxide [11]. Furthermore, modulation of Foxo1 expression inhibits inward sodium currents [12]. Thus, the potential relationships between *PDK1* signaling through Foxo1 and the function of Nav1.5 in the dysregulation of electrophysiological basis need further investigation.

In the clinic, arrhythmia is one of the primary cardiovascular events in patients with heart failure, and ventricular arrhythmias can lead to sudden death [13]. Specially, the cardiac sodium channel (*SCN5A*) mutations that reduce peak sodium current (such as mutations linked to Brugada syndrome) and QTc prolongation in patients with LQT3, or overlap syndrome. In animal experiments, *PDK1* knockout mice die suddenly of heart failure; however, the potential electrophysiological basis lead to the sudden death has not been determined. Thus, the relationship between *PDK1* and dysregulation of electrophysiology remains unclear. To understand the function of *PDK1* in sodium channel activation and the dysregulation of electrophysiological basis, we prepared a conditional deletion of *PDK1* in mouse cardiomyocytes through Cre-mediated excision. Using this conditional *PDK1*-deletion mouse, and using neonatal rat cardiomyocytes to exclude the possible impact of heart failure partly, we tested the hypothesis that *PDK1* regulates sodium channel activation in cardiomyocytes via the *PDK1*-Foxo1 pathway, thus facilitating the potential mechanism in the development of dysregulation of electrophysiology.

## Materials and Methods

### Generation of Conditional *PDK1* Knockout Mice

**Fig 1:**
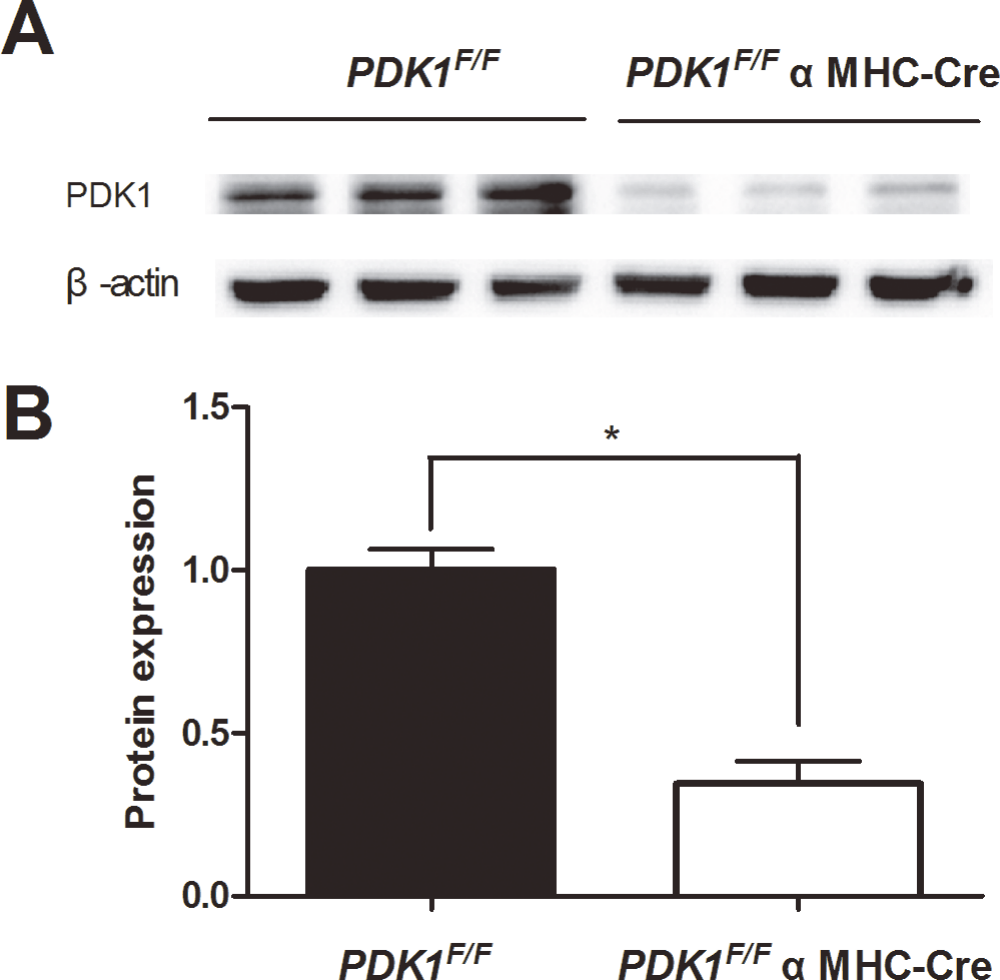
Generation of cardiomyocyte-specific *PDK1* deletion mice. A) Western blot analysis of *PDK1* expression in hearts from 6 representative control *PDK1* ^*F/F*^ mice and 6 representative *PDK1* ^*F/F*^ αMHC-Cre knockout mice. β-actin was tested as a loading control. B) The mean +SEM values from each group were normalized to 1.0 for the control mice. *P <0.05

Standard tissue-specific “knockout” approaches using the “Cre-Loxp” system were used to delete *PDK1* in the myocardium. *PDK1*-floxed mice were kindly provided by Dr. Zhongzhou Yang (Ministry of Education Key Laboratory of Model Animal for Disease Study, Nanjing University, China) [14]. In brief, *PDK1*-floxed mice (*PDK1* ^F/F^) were crossed with αMHC (α-myosin heavy chain)-Cre mice to delete *PDK1* in cardiomyocytes [9]. The deletion of *PDK1* in the myocardium was confirmed by Western blot analysis (Fig. 1). *PDK1* ^F/F^ αMHC-Cre mice were used in this study, and *PDK1* ^*F/F*^ littermates without the αMHC-Cre transgene were housed as control. This study was approved by the ethical committee of the First Affiliated Hospital of Nanjing Medical University, and all animal experiments were conducted under the guidelines on humane use and care of laboratory animals for biomedical research published by the National Institute of Health (No. 85-23, revised in 1996).

### ECG

ECG (Lead II) was performed under anesthesia (ketamine hydrochloride, 10 mg/kg, intraperitoneally) and continuously recorded by the RM6240B multiple channel physiological signal collecting and processing system (Chengdu Instrument Factory, Chengdu, China). The heart rate (HR), QRS and QT durations were measured. The QTc was calculated by Bazett’s formula, where QTc = QT/√RR.

### Mouse Cardiomyocyte Isolation

Single ventricular myocytes were obtained by enzymatic dissociation as previously described [15]. In brief, 8 weeks and 11 weeks of age mice were sacrificed by cervical dislocation, and the hearts were quickly placed in Tyrode’s solution containing 130 mM NaCl, 5.4 mM KCl, 1.8 mM NaH_2_PO_4_, 1.2 mM MgSO4, 5 mM HEPES, 20 mM taurine, 10 mM glucose, and 1.8 mM CaCl_2_ (pH 7.3) at 4°C. Then the aortas were quickly cannulated onto a modified Langendorff perfusion system for coronary perfusion. The hearts were perfused with Tyrode’s solution at 36°C for 1 min, followed by Ca^2+^ free Tyrode’s solution at a reduced rate of 3 ml/min. After 5 minutes, Ca^2+^ free Tyrode’s solution containing 150 U/ml Collagenase Type II (Worthington, Lakewood, USA) was applied for another 12 minutes until the hearts were palpably flaccid. Next, the ventricles were minced and gently agitated, and the cardiomyocytes were placed in KB solution containing 70 mM L-glutamic acid,50 mM KOH, 40 mM KCl, 20 mM taurine, 20 mM KH_2_PO4, 3 mM MgCl_2_, 0.5 mM EGTA, 10 mM D-glucose, and 10 mM HEPES (pH 7.3) at room temperature for patch clamp experiments. All chemicals used above were purchased from Sigma (Sigma Chemical Co., St. Louis, USA), except Collagenase Type II.

### Ventricular Myocyte Culture

Neonatal rat cardiomyocytes were prepared from 1–3 day old Sprague Dawley rats by enzymatic digestion. Hearts were excised and placed in sterile PBS solution. After connective tissue and blood were removed, the ventricles were minced and subjected to 8 min enzymatic digestion using serial 0.06% collagenase II and 0.08% pancreatin (both were purchased from Gibco, Thermo Fisher Scientific Inc., Waltham, USA) digestion. Cells were pre-plated on 10 cm petri dishes for 2 h to remove fibroblast cells and then cultured for 24 h in 10% (v/v) fetal bovine serum in Dulbecco’s modified eagle’s medium (high glucose) (Gibco) and 1% penicillin-streptomycin solution (Gibco) at 37°C in a humidified incubator with 5% CO_2_. After 24 h the media was replaced with 3% fetal bovine serum containing media.

### Cardiac Sodium Current Recording

In patch clamp experiment, 8 weeks and 11 weeks *PDK1* ^*F/F*^ αMHC-Cre mice were used as experimental group, and *PDK1* ^*F/F*^ mice in the same age were used as control group. Cells were transferred to a chamber (Warner Instrument Co, USA) and perfused with bath solution (5 mM NaCl, 130 mM choline chloride, 1.8 mM CaCl_2_, 1.8 mM MgCl_2_, 1 mM TEA-Cl, 10 mM HEPES, and 10 mM glucose, pH 7.4 with Tris base) at a constant rate of 1 ml/min. 200 μM NiCl2 and 1 μM nisoldipine were applied to block T- and L- type calcium currents. Beside, low Na+ concentration is conducive to control Na+ current recording [16]. Pipettes (Sutter Instrument Co, Novato, USA) were pulled by the Pipette Puller Model P-1000 (Sutter instrument Co, Novato, USA). Current recording was performed with an Axopatch 200B amplifier (Molecular Devices, Union City, CA) and a Digidata 1440A (Molecular Devices, Union City, CA). Pipettes were filled with pipette solution (130 mM CsOH, 130 mM Aspartic acid, 2.5 mM NaCl, 1 mM CaCl_2_, 1 mM MgCl_2_, 10 mM EGTA, 10 mM TEA-Cl, and 10 mM HEPES, pH 7.2 with CsOH) and a resistance of 1–2 MΩ was applied. Data expressing large series resistance (> 10 MΩ) were rejected. All chemicals used above were purchased from Sigma (Sigma Chemical Co., St. Louis, USA).

Sodium currents were evoked by a voltage-clamp protocol [16]. Current recordings from adult mouse cardiomyocytes and neonatal rat cardiomyocytes were established using a whole-cell patch clamp technique at room temperature (20–22°C) for at least 5 min. The cell membrane capacitance was then calculated by integrating the capacitive transient current in response to a 5-mV depolarizing pulse. The ionic current density (pA/pF) was calculated from the current amplitude of cell capacitance. Gating kinetics of sodium currents were recorded.

### Western Blot Analysis

Cardiac ventricular tissue and cultured cardiomyocytes were homogenized with a Kinamatica homogenizer (Bio-Gen Senes, Serial No.02-0145) in lysis buffer. After centrifugation for 15 min at 4°C at 14000g, the supernatants were collected and the protein concentrations were measured using the bicinchoninic acid (BCA) method. Nucleoprotein extracts were prepared using NE-PER Nuclear and Cytoplasmic Extraction Reagents (Pierce, Rockfort, IL, USA). A total of 30–50 μg protein was electrophoresed in SDS–polyacrylamide gels (Invitrogen) and transferred to polyvinylidene difluoride membranes (Roche Diagnostics GmbH, Germany). After blocking with 5% non-fat milk, membranes were incubated with specific primary antibodies overnight. Then the membranes were washed three times with TBST buffer, incubated with the corresponding horseradish peroxidase-conjugated secondary antibody for 1 h and developed onto a Molecular Imager (BIO-RAD Laboratories, Hercules, USA) using the enhanced chemiluminescence (ECL) system. Primary antibodies were as follows: FOXO1 (#2880, Cell Signaling, Danvers, USA), Akt (pan) (#4691, Cell Signaling), phospho-FoxO1 (Thr24) (#9464, Cell Signaling), phospho-Akt (Thr308) (#13038, Cell Signaling), β-actin (#4967, Cell Signaling), anti-*SCN5A* (AV35542, Sigma Chemical Co., St. Louis, USA). Three bands for each protein represented ventricular tissue from three different hearts in experimental or control group respectively. The protocol of western blotting data analysis was previously described [17].

### Drugs

For drugs used in patch clamping experiments, cells were maintained in culture media and final concentration of 100 nM of the *PDK1* inhibitor GSK233447 (Sigma Chemical Co., St. Louis, USA) [18], or the Akt inhibitor MK2206 (Selleck, Houston, USA) [19], or the Foxo1 inhibitor AS1842856 (Merck Millipore, Billerica, USA) [20,21] or a combination of inhibitors were applied in cell culture media for 48 h. All these drugs mentioned above were dissolved in dimethyl sulfoxide (DMSO), with the final concentration of dimethyl sulfoxide is 0.2% in our experiments.

### Statistics

Results are presented as mean ± SE. Statistical comparisons were made using the student’s t test. Differences were considered statistically significant if *P*<0.05.

## Results

### The ECG Profile Is Altered in *PDK1*-deletion Mice

**Fig 2:**
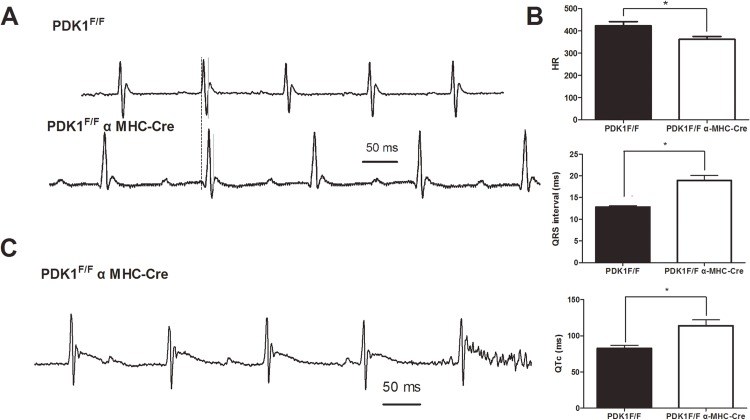
Electrocardiography of *PDK1* deletion mice. A) ECGs in lead II Representative ECG traces are shown for a control *PDK1* ^*F/F*^ mouse (top) at 8 weeks and a *PDK1* ^*F/F*^ αMHC-Cre knockout mouse (bottom) at 8 weeks. B) Slower heart rate and prolongation of QRS and QTc intervals in *PDK1* ^*F/F*^ αMHC-Cre mice at 8 weeks (n = 6). *P <0.05. C) Abnormal conduction in mice at 11 weeks. The trace is representative of results from 3 out of 4 *PDK1*>^*F/F*^ αMHC-Cre knockout mice.

To examine the mechanism of dysregulated electrophysiology in *PDK1* knockout mice, we performed ECGs of *PDK1* ^F/F^ αMHC-Cre knockout mice (n = 6) and control *PDK1* ^F/F^ littermates (n = 6) without the αMHC-Cre transgene. We measured the HR, QRS and QTc intervals, which are closely related to the onset of arrhythmias. After 8 weeks of deletion, HR was lower in *PDK1* knock mice (362.22±12.69 vs. 422.31±20.10, *P*<0.05), and both the QRS intervals (12.81±0.30 ms vs. 18.93±1.17 ms) and the QTc duration (82.69±4.08 ms vs. 113.91±8.20 ms) were significantly longer in the *PDK1*-deletion mice than the control mice (*P*<0.05; Fig. 2A and 2B). An abnormal conduction was found in 3 of 4 mice with *PDK1* deletion at 11 weeks, but no anomalies occurred in the control *PDK1* ^F/F^ mice (Fig. 2C). These results indicated the association between *PDK1* and dysregulated electrophysiology.

### *PDK1* Deletion Reduces the Sodium Current Density and Mediates a Slight Change in the Sodium Channel Kinetics

**Fig 3:**
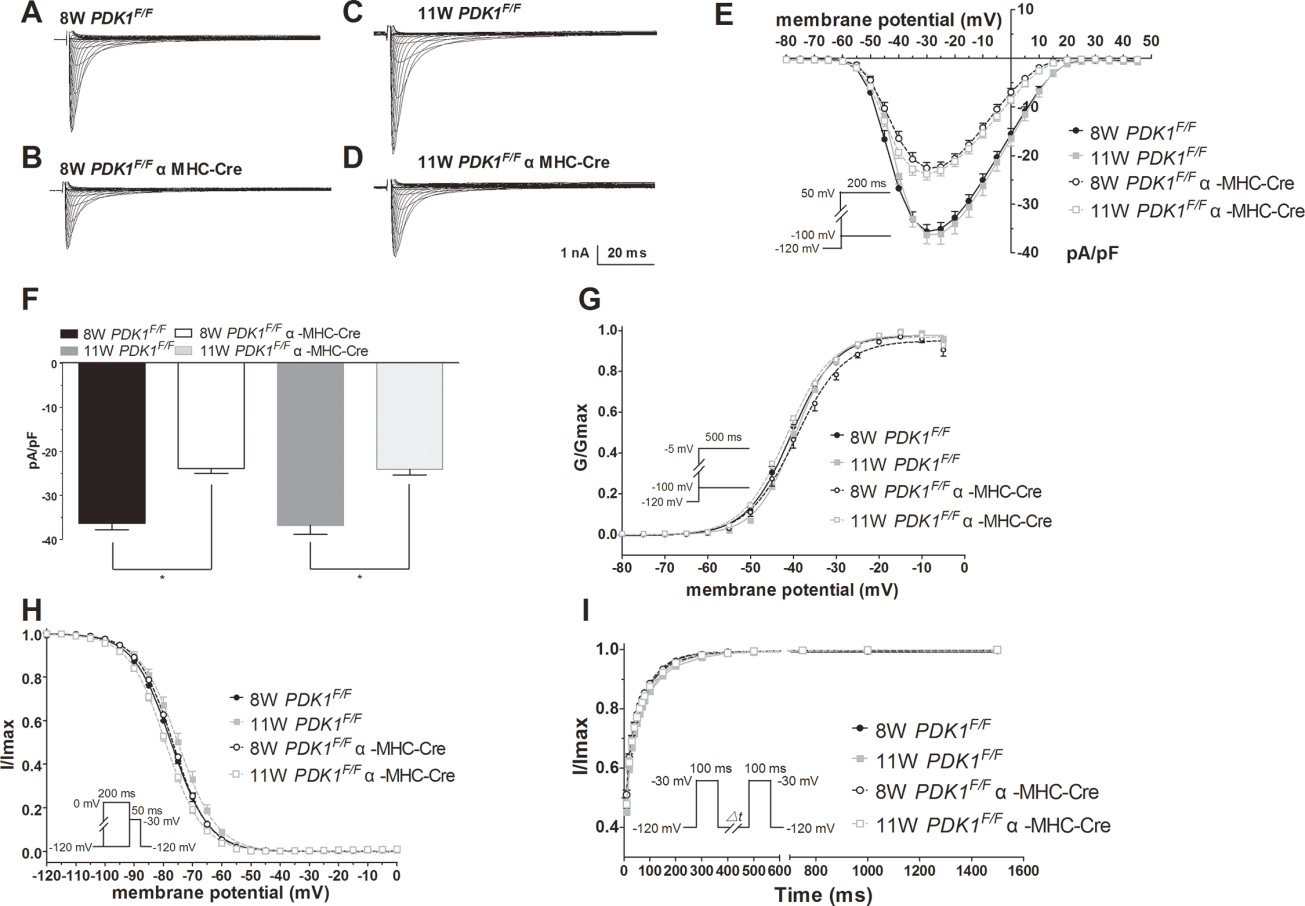
Patch clamping in cardiomyocytes from mice lacking *PDK1*. (A-D) Sodium current recording in *PDK1* ^*F/F*^ control and *PDK1* ^*F/F*^αMHC-Cre knockout mice at 8 and 11 weeks. E) Current-voltage relationship for mice at different weeks. F) Suppression of sodium current in mice lacking *PDK1*. G) Voltage dependence of activation. H) Voltage dependence of inactivation. I) Time-dependent recovery for sodium channel. (For cardiomyocytes, n = 13 to 32; for cells from mice, n = 2 to 3 for each group) *P <0.05

To determine the underlying mechanisms of ECGs alteration, we recorded sodium currents using the whole-cell patch clamping technique. Current traces of cells from *PDK1* ^*F/F*^ *αMHC-Cre* and *PDK1* ^*F/F*^ mice were determined using 5 mV increment test pulse from −100 mV to 45 mV at a holding potential of −120 mV. At −30 mV, peak values of sodium currents were −23.86±1.10 pA/pF in *PDK1* ^*F/F*^ *αMHC-Cre* cells (Fig. 3A) and −36.34±1.45 pA/pF in *PDK1* ^*F/F*^ cells (*P*<0.05, Fig. 3B), indicating a significant reduction of sodium current for *PDK1* ^*F/F*^ *αMHC-Cre* cells. Similar results were observed in the 11 weeks olds mice (−24.11±1.23 pA/pF in *PDK1* ^*F/F*^ *αMHC-Cre* cells vs. −36.76±2.07 pA/pF, *P*<0.05) in *PDK1* ^*F/F*^ cells. (Fig. 3C-3F). These results suggest that the sodium current amplitude is decreased by about 33% in *PDK1* ^*F/F*^ *αMHC-Cre* mice.

Defects in cardiac sodium channels can disrupt channel gating and cause electrical abnormalities. To further investigate whether current kinetics are affected by *PDK1* deletion, the activation, inactivation and recovery from inactivation curves were recorded. The steady-state activation and inactivation of sodium current were fitted by Bolzmann function, and the recovery from inactivation curves were fitted using a double exponential equation. As shown in Fig. 3G-3I and Table 1, fitting parameters of steady-state activation were similar in age-matched mice, but the midpoint of voltage dependence was slightly left-shifted for *PDK1* ^*F/F*^ *αMHC-Cre* mice at 11 weeks. For *PDK1* ^*F/F*^ *αMHC-Cre* knockout vs. *PDK1* ^*F/F*^ groups at 11 weeks, the V_1/2_ for activation was −41.14±0.61 mV vs. −39.61±0.68 mV and the slope was 5.07±0.15 vs. 4.37±021. Slight changes were also observed in the inactivation curves at 11 weeks (−79.02±0.56 mV vs. −75.09±1.17 mV; p<0.05); however, the slope of the Boltzmann curves did not change significantly. For recovery from inactivation, the same trend of variation between *PDK1* ^*F/F*^ *αMHC-Cre* and *PDK1* ^*F/F*^ groups was detected. The τ_f_ and the τ_s_ were shortened in the *PDK1*-deletion vs. control cells both at 8 weeks and at 11 weeks, with statistical differences in the τ_f_ at week 8 and the τ_f_ at week 11 (Table 1). These results demonstrate that *PDK1*-deletion causes a decrease in sodium current amplitude that may be explained in part by electrical abnormalities.

**Table 1:** Biophysical parameters for sodium channel kinetics in mice with different age.

	Voltage dependence of activation			Voltage dependence of inactivation			Recovery from inactivation		
	V_1/2_ (mV)	K	n	V_1/2_ (mV)	K	n	τ_f_ (ms)	τ_s_ (ms)	n
8W *PDK1* ^*F/F*^	−40.44±0.68	4.82±0.14	27	−77.21±0.67	−6.14±0.07	28	5.15±0.28	51.51±2.40	26
8W *PDK1* ^*F/F*^ αMHC-Cre	−38.37±0.95	4.61±0.21	35	−76.65±0.85	−5.61±0.0.05	34	4.36±0.19*	49.92±1.72	32
11 W *PDK1* ^*F/F*^	−39.61±0.68	4.37±0.21	16	−75.09±1.17	−6.19±0.08	14	6.22±0.17	69.32±1.47	13
11W *PDK1* ^*F/F*^ αMHC-Cre	−41.14±0.61	5.07±0.15	28	−79.02±0.56*	−6.17±0.11	20	5.98±0.44	60.04±1.93*	14

*P<0.05, compared with same week.

### Impaired *PDK1*-Foxo1 Pathway Activation Leads to Reduced Sodium Channel Expression in *PDK1*-Deletion Mice

**Fig 4:**
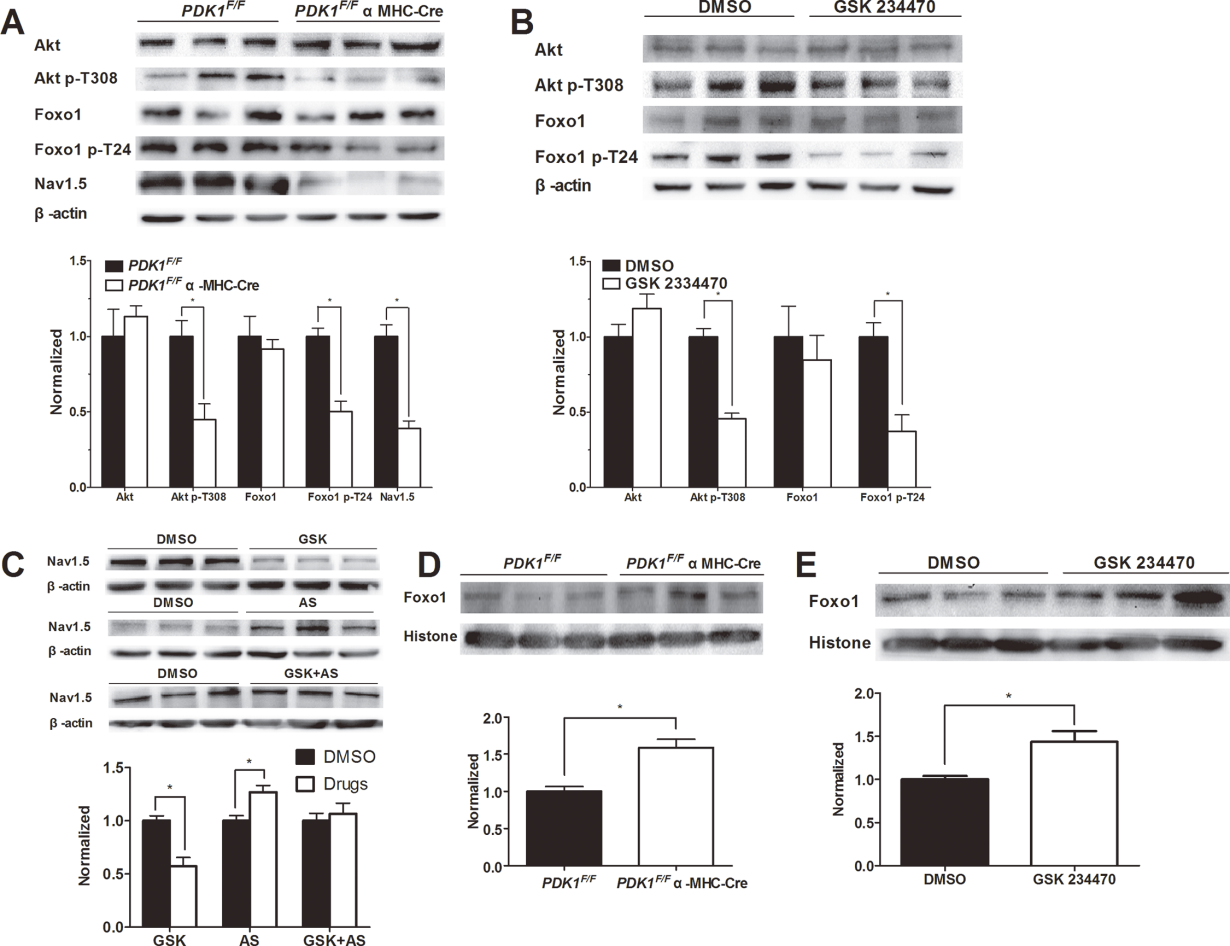
Western blot analysis of the *PDK1* signaling pathway. A) Western blot analysis was performed to assess levels of the indicated of proteins and phosphoproteins in *PDK1* ^F/F^ control and *PDK1* ^F/F^ αMHC-Cre knockout mice. B) Expression of total and phosphorylated Akt and Foxo1 was assessed after treatment of cardiomyocytes from neonatal rats with vehicle control (DMSO) or *PDK* inhibitor (GSK 234470). C) The expression of Nav1.5 in rat cardiomyocytes was assessed after treatment with DMSO, GSK 234470 (GSK), the Foxo1 inhibitor AS 1842856 (AS) or a combination of the two drugs. D-E) Nuclear Foxo1 expression was assessed for cardiomyocytes isolated from control and *PDK* deletion mice (D) or neonatal rat cardiomyocytes before and after treatment with *PDK* inhibitor (E). β-actin was tested as a loading control for total cellular protein levels, and histone H3 was tested as a loading control for nuclear protein levels. The mean +SD values from each group were normalized to 1.0 in the control mice. All drugs were dissolved in DMSO and added into cell culture media for 48h before the experiments. In each group, three bands of proteins represented three different hearts. *P <0.*05*.

To investigate the effects of *PDK1* deletion on the Akt/Foxo1 pathway and the potential downstream effects on sodium channel expression, proteins were extracted from ventricle tissues of control *PDK1* ^*F/F*^ and *PDK1* ^*F/F*^ αMHC-Cre knockout mice. There were no obvious differences in the protein expression levels of Akt and Foxo1; however, increased levels of phosphorylation of Akt on Thr308 and Foxo1 on Thr24 were observed upon *PDK1* deletion (Fig. 4A). Consistent with the results of patch clamping, markedly reduced expression of Nav1.5 in the *PDK1* knockout group was also observed.

To verify the activation of Akt and Foxo1 phosphorylation by *PDK1*, we applied the *PDK1* inhibitor GSK 2334470 to isolated cardiomyocytes from rats. Similar to the results from the *PDK1* knockout mice, no obvious change in the total protein expression of Akt and Foxo1 was found between the GSK 234470 group and the DMSO control group, but the levels of phosphorylation of these proteins were decreased in the GSK 2334470 group (Fig. 4B). GSK 2334470 also decreased the expression of Nav1.5, though the Foxo1 inhibitor AS 1842856 had the opposite effect on Nav1.5 expression and could counteract the effect of GSK 2334470 when the two drugs were used in combination (Fig. 4C). Because Foxo1 is an inhibitor of the *PDK1* pathway that is inactivated by phosphorylation [12], these results are consistent with the positive role of the *PDK1* pathway in regulating the expression of a sodium channel in cardiomyocytes.

Because phosphorylation affects the subcellular distribution of Foxo1 [11], we isolated nuclear proteins from control and *PDK1* deletion cells and assessed the levels of Foxo1 relative to the nuclear protein. The nuclear Foxo1 levels were increased in *PDK1* knockout cells compared to control cells (Fig. 4D). Furthermore, the levels of Foxo1 were increased by GSK 234470 (Fig. 4E). Collectively, these results support a model by which *PDK1* deletion inhibits the activity of the Akt pathway, leading to increased Foxo1 activity and consequently, reduced Nav1.5 expression.

### *PDK1* and Foxo1 Specific Inhibitors Regulate Sodium Channel Activation

**Fig 5:**
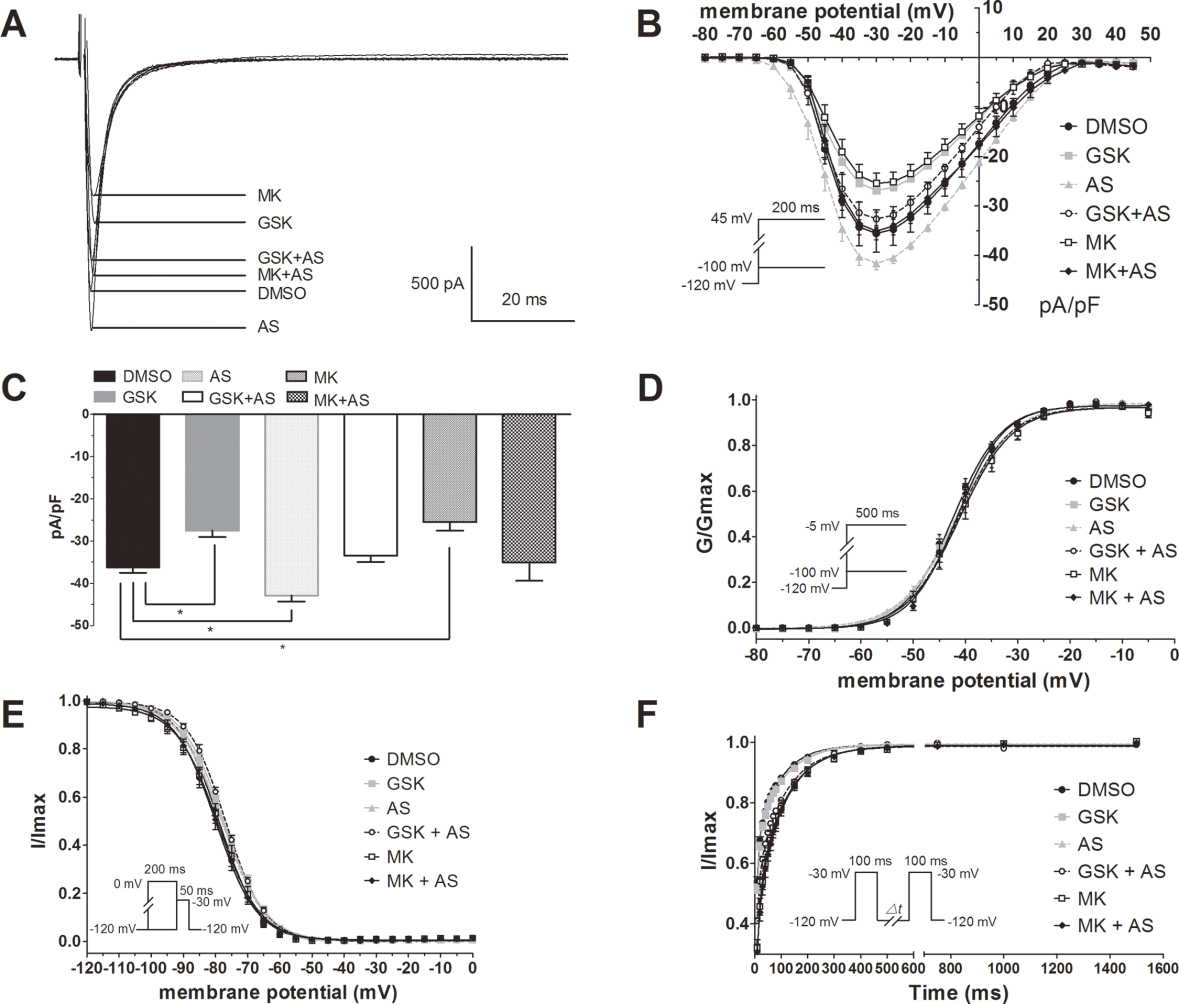
Patch clamping results in neonatal rat cardiomyocytes. A) Sodium current recording in neonatal rat cardiomyocytes exposed to different inhibitors alone or in combination. B) Current-voltage relationship for cells incubated with drugs for 48 h. C) Changes in sodium current in cells after drug treatment. D) Voltage dependence of activation. E) Voltage dependence of inactivation. F) Time dependent recovery for sodium channel. (For cardiomyocytes, n = 6 to 24 per group of control experiments). GSK (GSK 234470) is a *PDK1* inhibitor, MK (MK 2206) is an Akt inhibitor, and AS (AS 1842856) is a Foxo1 inhibitor. All drugs were dissolved in DMSO and added into cell culture media for 48h before the experiments,*P<0.*05*

Lack of *PDK1* in mouse cardiac muscle has been shown to be associated with less thickening of the ventricular wall and lead to heart failure [9]. To rule out an impact on chronic left ventricular function decline, neonatal rat cardiomyocytes were treated with the *PDK1* inhibitor GSK 2334470, the Akt inhibitor MK2206, the Foxo1 inhibitor AS 1842856, or a combination of drugs, and the effects on sodium channels were investigated using the patch clamp technique. The amplitude of the current in untreated cells (−36.23±1.25 pA/pF) was suppressed by treatment with either GSK 2334470 (−27.52±1.49 pA/pF) or MK 2206 (25.48±2.06 pA/pF) (*P*<0.05), supporting the positive role of *PDK1* and Akt (Fig. 5A-5C). Conversely, the amplitude of sodium current was increased after application of AS 1842856 (−42.85±1.51 pA/pF; *P*<0.05), which is consistent with the negative role of Foxo1. Furthermore, the sodium current had no obvious change when a combination of GSK 2334470 and AS 1842856 (−33.41±1.50 pA/pF) or MK 2206 and AS 1842856 (−35.09±4.29 pA/pF) were applied. These results verify the role of the *PKD1* pathway on sodium channel activation.

As a control, there were no obvious changes in the biophysical parameters of sodium currents for activation and inactivation in the presence of GSK 2334470, MK 2206, AS 1842856 or a combination of drugs (Fig. 5D-5E and Table 2). However, the time of recovery was delayed when MK 2206 or a combination of drugs were applied. These results verify that the *PDK1*-Foxo1 pathway regulates the amplitude of the sodium current without consistent impact on the sodium channel kinetics.

**Table 2:** Biophysical parameters for sodium channel kinetics in the presence of different drugs.

	Voltage dependence of activation			Voltage dependence of inactivation			Recovery from inactivation		
	V_1/2_ (mV)	K	n	V_1/2_ (mV)	K	n	τ_f_ (ms)	τ_s_ (ms)	n
DMSO	−42.25±0.85	4.20±0.14	19	−79.02±0.62	−6.06±0.12	21	4.86±0.27	62.23±2.72	19
GSK 234470	−41.23±0.96	4.75±0.19	21	−77.37±0.85	−6.03±0.11	23	5.39±0.36	70.21±3.66	21
AS 1842856	−41.91±1.06	4.46±0.15	24	−78.24±0.88	−6.13±0.12	25	5.61±0.39	66.85±3.41	24
GSK 234470+AS 1842856	−41.33±1.34	4.56±0.21	7	−76.90±0.48	−5.99±0.29	6	6.04±0.26*	74.79±3.69*	6
MK 2206	−40.88±1.37	4.29±0.28	13	−79.36±1.16	−6.09±0.22	14	7.58±0.88*	70.12±6.33	11
MK 2206+ AS 1842856	−41.49±0.78	4.31±0.36	9	−79.95±1.15	−6.17±0.16	8	7.95±0.61*	70.28±3.08	8

*P<0.05, compared with DMSO (dimethyl sulfoxide).

All drugs were dissolved in DMSO and added into cell culture media for 48h before the experiments.

## Discussion

In the present study, we have demonstrated for the first time that *PDK1* participates in the dysregulation of electrophysiological basis by regulating the expression of Nav1.5. This effect is likely to be mediated through the regulation of the phosphorylation and expression of the *PDK1*-Foxo1 pathway.

Previous studies have reported that Tamoxifen-inducible and heart-specific disruption of *PDK1* in adult mice causes severe and lethal heart failure [6,9,14]. It is well known that arrhythmia is one of the most common phenomenons in patients with heart failure [22]. However, little is known about the dysregulation of electrophysiological basis by *PDK1* knockout. In this study, differences in ECG and sodium channel parameters were found in *PDK1*-deletion mice, and the effects were shown to be induced via the *PDK1*-Foxo1 pathway. The finding may be the underlying mechanisms between *PDK1* and the voltage-gated sodium channels are responsible for electrophysiological abnormalities in the heart. Lu et.al [23] reported a mechanism for QT prolongation that involved an increase in persistent sodium current (late current) caused by defective PI3K signaling, however, their previous work [10] suggested that PI3K inhibitor decreased multiple ion current, including peak Na+ current. Much evidence has shown that a decline or dysfunction of sodium currents is responsible for QT interval prolongation or conduction blockade [24,25]. Especially, Remme et.al [26] and Korkmaz et.al [27] reported that downregualtion of sodium current lead to lower heart rate and conduction dysfunction, but kinetics of voltage-dependent activation and inactivation had no obvious difference. In current study, we found that the QRS and QTc intervals are prolonged in *PDK1*-deletion mice. A change in ventricular conduction can be manifested as prolongation of the QRS interval on the ECG, and drug-induced effects on ventricular conduction are often associated with block of cardiac sodium current. Heath et al. [28] found that QRS interval prolongation (∼10–20%) observed in either preclinical or clinical studies with sodium channel inhibitor. In addition, an earlier report by Cordes et al. [29] indicated that blocking sodium current was sufficient to produce QRS widening. Previous findings also predicted that small reductions in sodium current might affect intraventricular conduction [30]. While the relationship between changes in conduction velocity and QRS widening were not determined. Our findings demonstrated that reductions in cardiac sodium currents by *PDK1* knockout might affect intraventricular conduction. In our study, the results of patch clamping experiments indicated that the density of sodium current was reduced by about 33% in *PDK1* knockout mice. In addition, the steady-state inactivation and activation of the currents were minimally changed in the group with heart failure compared to the control group. The phenomenon is very similar to Remme’s [26] and Korkmaz’s [27] work. On the contrary, our results suggest that activation and inactivation curves are slightly left-shifted. Thus, further experiments to elucidate the relationship between the *PDK1* signaling pathway and sodium channel are needed.

*PDK1* phosphorylates Akt on Thr308, and phosphorylation of Thr308 is decreased in mice lacking the *PDK1* gene [8]. Furthermore, the PI3K/PTEN signaling pathway, which is upstream of *PDK1* and Akt, is involved in a wide variety of diseases including myocardial hypertrophy and contractility, heart failure, and preconditioning[31]. We have verified that Akt phosphorylation is reduced in *PDK1* knockout mice and in isolated rat cardiomyocytes treated with GSK 2334470, a *PDK1*-specific inhibitor that does not suppress the activity of 93 other protein kinases including 13 AGC-kinases [18]. Additionally, our results show that Foxo1 phosphorylation is reduced by *PDK1* deletion or inhibition. Similar reduction of Foxo1 phosphorylation was observed in the pancreatic islets of βPdk1 -/- mice[32]. Furthermore, cells lacking PTP non-receptor type 12 (PTPN12), which is an upstream mediator of *PDK1*, are defective in the activation of Foxo1/3a[33]. Foxo1 binds the promoter region of *SCN5A* [34], and exogenous Foxo1 expression decreases *SCN5A* promoter activity in HL-1 cells [11]. Previous investigation has shown that Foxo1 phosphorylation promotes its translocation from nucleus to cytoplasm, which relieves its ability to negatively regulate *SCN5A* [12]. Consistently, we observed increased nuclear Foxo1 expression levels in *PDK1*-deleted cells, which may explain decreased Nav1.5 levels. Our results are consistent with the possibility that Nav1.5 expression is downregulated by *PDK1* deletion via the suppression of phosphorylation of Akt and Foxo1.

In this study, the results suggested that a potential association of *PDK1* down-regulation and decreased cardiac sodium current destiny, resulting in the sudden cardiac death in mice with *PDK1* deletion. However, little is known on how reduction in peak sodium current caused death in these mice. But mutations in *SCN5A* have been previously linked to Brugada syndrome, conduction disturbances, sick sinus syndrome and dilated cardiomyopathy; most of these are associated with biophysical properties consistent with “loss of function” phenotype (reduced cardiac sodium current/function) [35]. Clinically, patient with Brugada syndrome which reduces peak sodium current is well-known to cause cardiac arrhythmias and some of these mutations also cause dilated cardiomyopathy [36,37]. Patients who carried those mutations had increased mortality and a high risk for sudden arrhythmic death [38]. Therefore, the *PDK1* deletion mice and Brugada syndrome had high similarity phenotypically and functionally, it is a reasonable assumption that PDK1 deletion mice shown a reduction in peak sodium current and that this may be a cause of sudden cardiac death, through mechanisms that have been described for the Brugada Syndrome. We would further speculate that *PDK1* may be a candidate gene for the Brugada syndrome. These need to be verified genetically in patients with Brugada syndrome. Thus, PI3K-*PDK1* signaling pathways may provide critical insights into arrhythmogenesis associated with “loss of function” phenotype.

On the other hand, it is not clear whether the decline occurs independently of heart failure. Zicha et al. reported a maximum density of sodium current reduction of about 33.6% in the dog with heart failure [39]. To exclude the possible impact of heart failure and to support the association of the *PDK1* pathway with sodium current and sodium channel expression, *PDK1* signaling pathway specific inhibitors were applied to neonatal rat cardiomyocytes. In patch clamping assays, neonatal rat cardiomyocytes treated with *PDK1* or the Akt inhibitor for 48 h decreased the sodium current from −36.23±1.25 pA/pF to −27.52±1.49 pA/pF (*P*<0.05) or −25.48±2.06 pA/pF (*P*<0.05), which was similar to the in vivo results of *PDK1* deletion. Conversely, the peak sodium current increased to-42.85±1.51 mV (*P*<0.05) when the Foxo1 inhibitor AS1842856 was applied, which is consistent with its inhibitory role in expression of Foxo1 [21]. Furthermore, AS1842856 appeared to counteract the decrease promoted by the other two drugs, and no variations the channel activation or inactivation kinetics were detected, which suggests that Foxo1, one of the *PDK1* downstream factors, involve in the regulation of sodium channel. Our results demonstrate that the expression of Nav1.5, the product of the *SCN5A* gene, is markedly reduced in *PDK1* deletion mice via the *PDK1*-Foxo1 pathway. These findings are supported by the use of *PDK1*, Akt and Foxo1 inhibitors.

*PDK1* regulates phosphoinositide PI3K and activates a number of AGC kinases, including Akt, p70 ribosomal S6 kinase (p70S6K), and SGK1. Much evidence suggests that SGK1 stimulates a variety of ion channels [3], including Nav1.5, and that the ubiquitin ligase Nedd4 may regulate SGK1 and its downstream effects on *SCN5A* [40]. However, the effect of SGK1 is modest, and lack of SGK1 does not fully disrupt the downstream effects [41]. In addition, small G proteins, molecular switches that control the activity of cellular and membrane proteins, regulate a wide variety of ion channels. In some cases, K-Ras and RhoA increase the activity of the epithelial sodium channel (ENaC) via PI3K and PI(4)P5-kinase signaling pathways[42]. Furthermore, Rho inhibits ether-a-go-go–related gene channel via a protein serine/threonine (S/T) kinase, whereas Rac stimulates the channel via a protein S/T phosphatase [43]. Whether small G proteins regulate Nav1.5 via the PI3K signaling pathway needs further research.

In conclusion, through current study we have discovered that the peak Na+ is reduced in *PDK1* KO mice and that this may be a cause of sudden death, through mechanisms that have been described for the Brugada Syndrome.

## Limitations

First, heart failure is a complex pathophysiological process, and multiple influence factors, including other AGC protein kinase family, involve in cardiac structure and electrophysiological remodeling. Furthermore, action potential and QT duration are dependent on balance between depolarizing inward and repolarizing outward current. It is unclear if the changes are related to the reduction in *PDK1* or due to some other changes associated with heart failure and outward potassium channels [10]. Second, we only made abnormal observations with body surface ECG, more reliable data, such as arrhythmias directly linked to the cause of sudden death, should be obtained using telemetry devices from *PDK1* ^*F/F*^ αMHC-Cre animals.
